# Surgical versus conservative management of minimally displaced (≤ 2 mm) pediatric lateral humeral condyle fractures: systematic review and meta-analysis

**DOI:** 10.1186/s12891-026-09555-w

**Published:** 2026-02-14

**Authors:** Feihu Li, Bin Dong, Chenjing Li, Binbin Xing

**Affiliations:** Department of Orthopaedics, Yuncheng Central Hospital Affiliated to Shanxi Medical University, YunCheng, ShanXi Province China

**Keywords:** Minimally displaced fracture, Lateral humeral condyle fracture, Conservative treatment

## Abstract

**Purpose:**

Lateral humeral condyle fracture (HLCF) is a common pediatric elbow injury. For minimally displaced fractures (≤ 2 mm), the optimal management strategy remains controversial. This systematic review and meta-analysis aimed to evaluate the feasibility and safety of conservative treatment for this specific subtype.

**Methods:**

A systematic search of multiple databases was conducted to compare conservative and surgical management of minimally displaced (≤ 2 mm) pediatric HLCF. The primary outcome was fracture healing, defined by the absence of secondary displacement during follow-up. Secondary outcomes included elbow joint function, periarticular hyperplasia, and malunion.

**Results:**

Twelve studies involving 1,022 children met inclusion criteria. Secondary displacement occurred in 18.4% of conservatively treated patients compared with 5.4% of surgically treated patients, indicating a higher risk of treatment failure with non‑operative management. Among fractures that ultimately healed, no significant differences were observed between conservative and surgical groups in elbow function, periarticular hyperplasia, or malunion.

**Conclusions:**

Even minimally displaced (≤ 2 mm) pediatric HLCFs may demonstrate mechanical instability during follow‑up. Conservative treatment should therefore be undertaken with caution, and close radiographic monitoring is essential to detect early displacement. Treatment decisions should integrate displacement magnitude, fracture stability, imaging findings, follow‑up reliability, and family preference.

**Supplementary Information:**

The online version contains supplementary material available at 10.1186/s12891-026-09555-w.

## Introduction

Lateral humeral condyle fracture (HLCF) is the second most common pediatric elbow fracture [1, 2]. Although minimally displaced pediatric fractures are often managed successfully with non‑operative approaches such as cast immobilization, HLCF—a distinct intra‑articular injury—can still lead to severe complications, even when initial displacement is minimal. Clinical outcomes following non‑operative treatment remain inconsistent: while Bast et al. reported excellent results in 93 minimally displaced cases treated conservatively [3], other studies have documented progressive displacement requiring surgical conversion, with rates ranging from 8.5% to over 10% [4, 5].

Compared with other pediatric fractures, HLCF carries a higher risk of instability and malunion due to its intra‑articular nature and vulnerability of the epiphyseal blood supply [6]. Derrick et al. reported early displacement in 14.9% and nonunion in 14.5% of conservatively treated patients, underscoring the limitations of relying solely on initial radiographic appearance [7]. Although classification systems such as Milch, Jacob, Song, and Weiss provide guidance [8–11]. consensus remains lacking for minimally displaced fractures (typically Type 1), whereas Types 2 and above are widely accepted surgical indications [12].

Importantly, the commonly used ≤ 2 mm displacement threshold—widely accepted as the criterion for conservative treatment in most pediatric fractures—may not reliably indicate true stability in HLCF. In long‑bone fractures, minimal displacement usually reflects an intact periosteal hinge and predictable healing. However, in HLCF, even ≤ 2 mm displacement may mask cartilaginous hinge disruption or mechanical instability, leading to secondary displacement or delayed healing [7, 13]. Thus, fractures classified as “minimally displaced” may not represent a biologically homogeneous group in this specific injury pattern.

Recognizing that secondary displacement reflects early mechanical instability and is commonly regarded as a marker of treatment failure, we conducted a systematic review and meta‑analysis comparing surgical and conservative outcomes, focusing on fracture healing as the primary outcome and elbow joint function, periarticular hyperplasia, and malunion as secondary outcomes.

## Methods

### Search strategy and selection criteria

This systematic review and meta-analysis was conducted in accordance with the PRISMA guidelines. Comprehensive searches of PubMed, MEDLINE, Embase, and the Cochrane Library were performed for the period 1950–2025. The protocol was registered with PROSPERO (registration number: CRD42023477290). A predefined search strategy combining MeSH terms and free-text keywords related to “pediatric,” “lateral humeral condyle fracture,”“minimally displaced,”“conservative treatment,” and “outcomes”was used. Boolean operators (AND/OR), truncation, and database-specific filters were applied. Full electronic search strategies are provided in Supplementary Material 1.

### Eligibility criteria

Studies were eligible if they met all of the following criteria: (1) pediatric patients under 16 years of age; (2) radiographically confirmed fracture displacement ≤ 2 mm post-injury; (3) reporting at least one therapeutic outcome, with a focus on conservative treatment; (4) outcomes including fracture healing or other clinical endpoints such as elbow joint function, anatomical changes at the healing site, abnormal healing, nerve injury, malunion.

Exclusion criteria included: (1) combined fractures of the same limb; (2) old fractures (≥ 3 weeks from injury); (3) lack of reported outcomes for conservative treatment efficacy.

### Data extraction and management

Two reviewers independently screened and extracted data, with discrepancies resolved by a third reviewer. Extracted variables included study design, patient demographics, treatment type, outcome measures, and follow-up duration.

### Outcome definitions

To ensure consistency across studies, outcome definitions were standardized prior to extraction. Secondary displacement was treated as the definitive indicator of treatment failure, and fracture healing was defined as the absence of displacement during follow‑up.

- Secondary displacement was defined as any increase in fracture gap or fragment translation during follow-up compared with initial post-injury radiographs. When displacement resulted in conversion from conservative management to surgical fixation, it was classified as treatment failure and designated as the primary outcome, as it reflects early mechanical instability during immobilization.
- Fracture healing was defined as the absence of secondary displacement, supplemented by radiographic evidence of bridging callus across ≥ 3 cortices and clinical findings such as absence of local tenderness and pain‑free motion.

### Fracture healing criteria and follow-up standardization

Fracture healing was evaluated using radiographic criteria (bridging callus across ≥ 3 cortices) supplemented by clinical findings (absence of local tenderness and pain-free motion). To address variability in follow-up duration, the longest available radiographic follow-up was used for healing-related outcomes.

### Outcome categories

Treatment outcomes were categorized as:

- *Primary outcomes*: fracture healing (absence of secondary displacement).
- *Secondary outcomes*: elbow joint function, nerve injury, hyperplasia after fracture healing.

### Data analysis and quality assessment

Dichotomous outcomes were synthesized using odds ratios (ORs) with 95% confidence intervals (CIs). For studies reporting conservative treatment alone, single-arm meta-analysis was performed to estimate pooled fracture healing rates as a secondary outcome. Statistical significance was defined as *P* < 0.05. When notable between-group differences were observed, absolute effects (risk difference and number needed to treat) were additionally calculated. Fisher’s exact test was applied in analyses with small event counts or imbalanced group sizes.

Heterogeneity was assessed using the I²statistic, with values > 60% indicating substantial heterogeneity. Publication bias was explored using funnel plots, although interpretation was limited by the small number of included studies.

Exploratory logistic regression was conducted on case‑level data to evaluate factors associated with secondary displacement, recognizing that these analyses are associative rather than predictive.

All analyses were performed using RevMan 5.4, with supplementary analyses conducted in Python. Study quality was evaluated using the Newcastle-Ottawa Scale.

## Results

### Search results

**Fig. 1:**
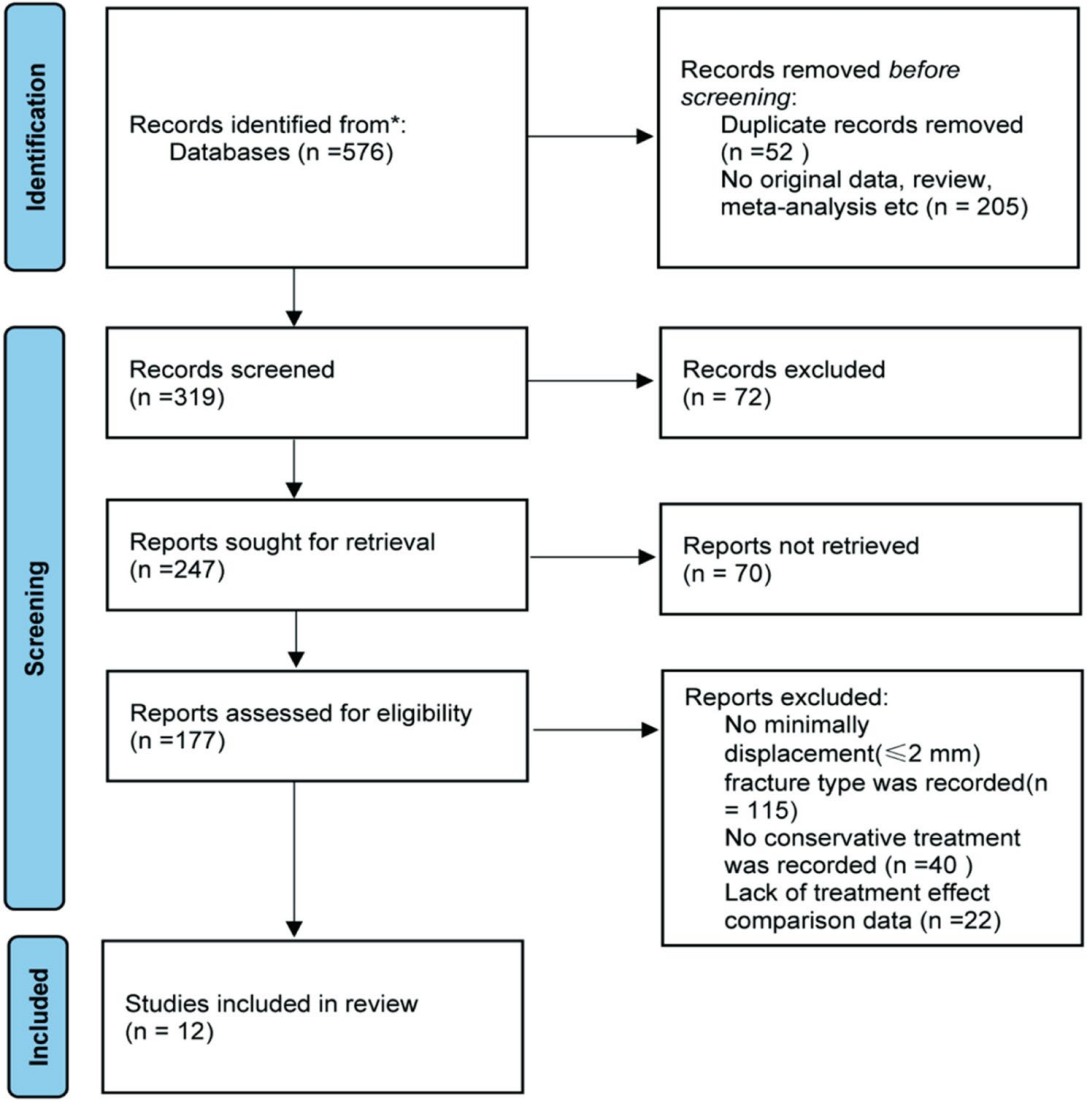
Flow diagram of the selection of reports for this meta-analysis

A comprehensive literature search identified 12 eligible studies comprising 1,022 pediatric participants (Fig. 1). Of these, 11 were retrospective and one was prospective. All articles were published in English. Table 1 summarizes the study characteristics and analyzed variables. Methodological quality was satisfactory across all included studies, with each achieving a Newcastle–Ottawa Scale score ≥ 6.

**Table 1:** Demographic data from the included literature

Authors, year	Study design	No.of Patients (female)	Group patients (conservation/operation)	Mean age (years)	Outcome indicators	Follow-up
Bast SC (1998) [3]	Retrospective	95 (30)	93/2	4 yrs	(1)(5)(6)(7)	19 weeks
S. thönell (1988) [14]	Retrospective	159	159/NR	6.2 yrs	(1)	NR
Finnbogason (1995) [5]	Prospective	118	112/6	6.3 yrs	(1)	NR
Franck L (2004) [13]	Retrospective	30(11)	17/13	6.1 yrs	(1)(3)(7)	12.3 months
Marcheix (2011) [15]	Retrospective	22(13)	7/15	4.6 yrs	(1)(5)(7)	15.7 months
Pirker (2005) [16]	Retrospective	51	51/NR	6 yrs	(1)	NR
Vigil J (2020 ) [17]	Retrospective	268(95)	243/25	7.6 yrs	(1)	6 months
Flynn (1975) [18]	Retrospective	31(10)	31/NR	7 yrs	(1)	NR
Kurtulmuş T (2014) [19]	Retrospective	27(7)	27/NR	4.5yrs	(1)	20-70months
O. Badelon (1988) [20]	Retrospective	23	16/7	NR	(1)(3)(5)	NR
Dustin. A (2019) [21]	Retrospective	139	114/25	4.6 yrs	(1)	2.4 ± 6.7 months
Zale C (2018) [4]	Retrospective	59(16)	59/NR	6.7 yrs	(1)	79.4 days

(1)fracture healing; (2) delayed healing rate; (3) malunion rate; (4) cast immobilization time; (5)elbow joint function evaluation; (6) Nerve injury; (7) hyperplasia after fracture healing

### Primary outcome and fracture healing

**Fig. 2:**
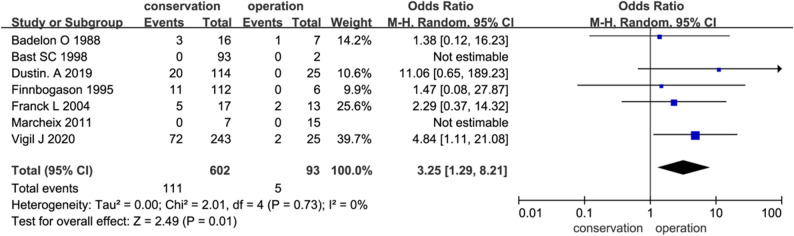
Comparison of fracture-healing failure (secondary displacement) between conservative and surgical treatment

Seven studies compared conservative and surgical management, while five evaluated conservative treatment alone. Secondary displacement—representing treatment failure—occurred in 18.4% of conservatively treated patients versus 5.4% of surgically treated patients. This difference was statistically significant, and Fisher’s exact test confirmed the association between conservative treatment and a higher risk of displacement (*P* < 0.01), with an estimated number needed to treat (NNT) of 8 to prevent one case of secondary displacement by choosing surgical fixation over conservative management (Fig. 2).

**Fig. 3:**
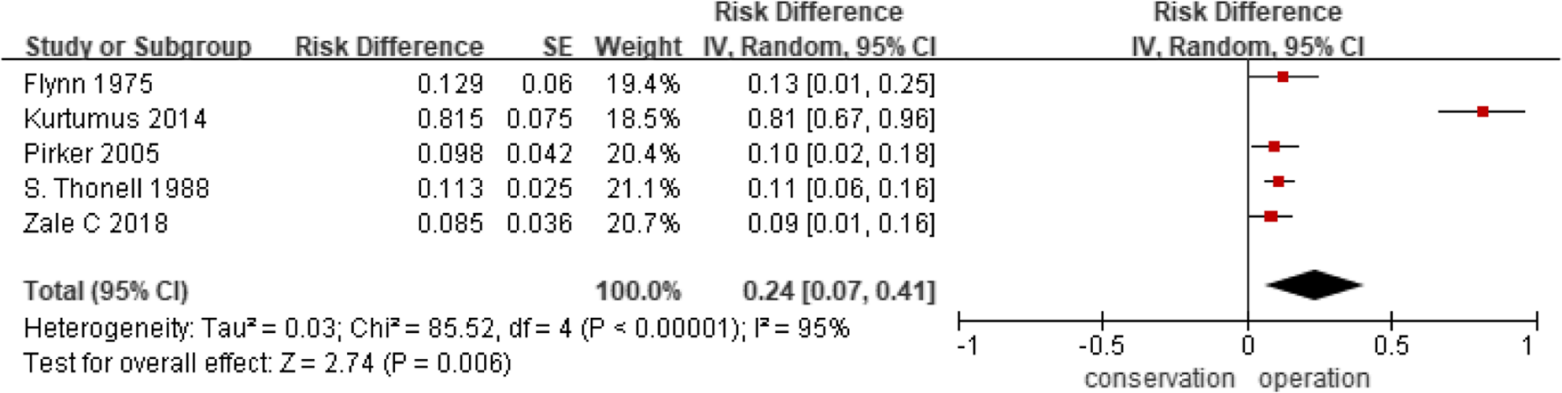
Fracture-healing rate in conservatively treated fractures

Among studies assessing conservative treatment alone, pooled analysis demonstrated a lower fracture‑healing rate as a secondary outcome (OR = 0.24; 95% CI: 0.07–0.41; *P* < 0.05; Fig. 3). Heterogeneity was substantial (I² = 95%) but resolved after exclusion of one outlier study, with the overall effect remaining significant, indicating that healing rates under conservative management were consistently lower in the remaining studies.

### Elbow joint function

**Fig. 4:**
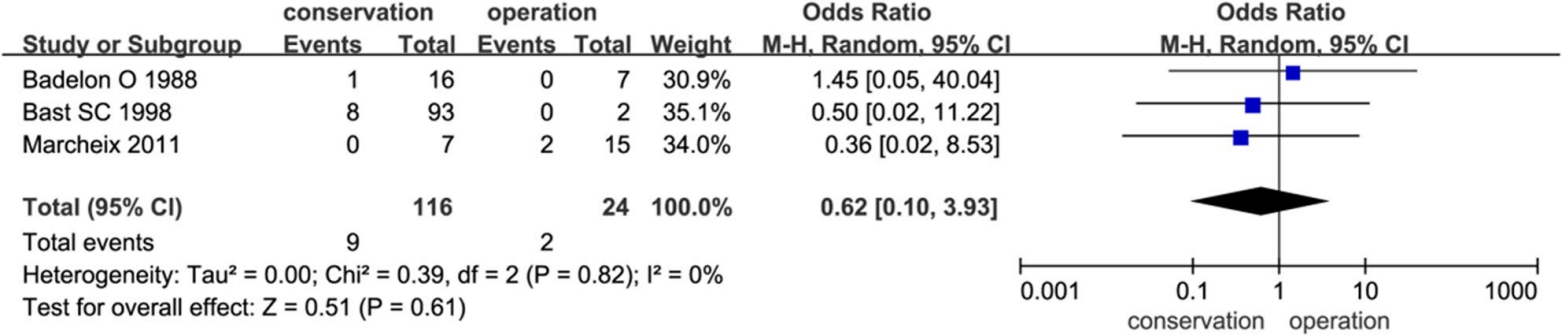
Comparison of elbow joint function after conservative and surgical treatment

Three studies assessed post‑treatment elbow function using the Dhillon scale (Fig. 4). No significant difference was observed between conservative and surgical groups (7.8% vs. 8.3%; OR = 0.62; 95% CI: 0.10–3.93; *P* = 0.61; I² = 0%).

### Hyperplasia after fracture healing

**Fig. 5:**
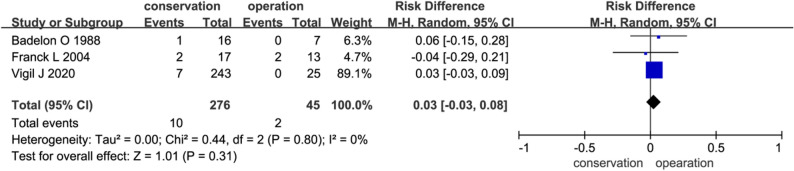
Comparison of bone hyperplasia after conservative and surgical treatment

Three studies reported periarticular hyperplasia (Fig. 5). Incidence was comparable between conservative and surgical groups (19.7% vs. 13.3%; OR = 1.14; 95% CI: 0.11–11.59; *P* = 0.91; I² = 58%).

### Malunion

**Fig. 6:**
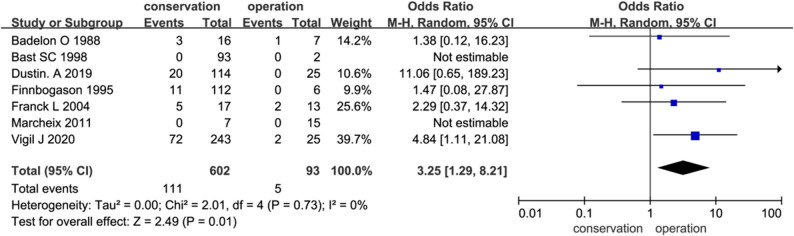
Comparison of malunion after conservative and surgical treatment

Three studies evaluated epiphyseal growth disturbances (Fig. 6). Malunion rates did not differ significantly (3.6% vs. 4.4%; OR = 0.03; 95% CI: -0.03 to 0.08; *P* = 0.31; I² = 0%).

### Risk prediction for fracture displacement

**Fig. 7:**
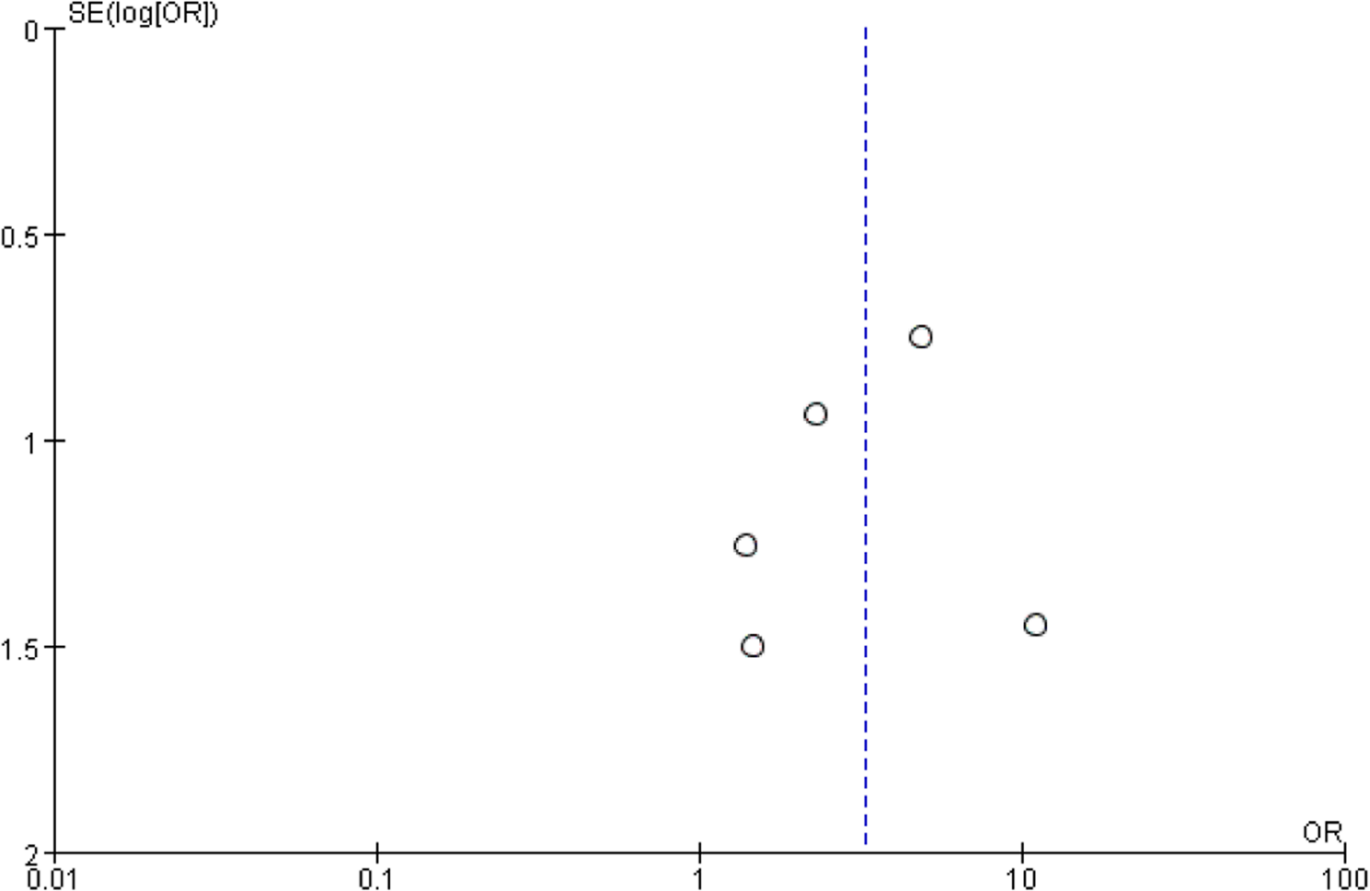
Funnel plot assessing publication bias in studies reporting displacement rates

Logistic regression models were constructed using case‑level data. ROC analysis showed limited predictive ability for the treatment‑only model (AUC = 0.58). Adding malunion or overgrowth yielded minimal improvement (AUC = 0.61), indicating weak discrimination (Fig. 7).

### Publication bias assessment

**Fig. 8:**
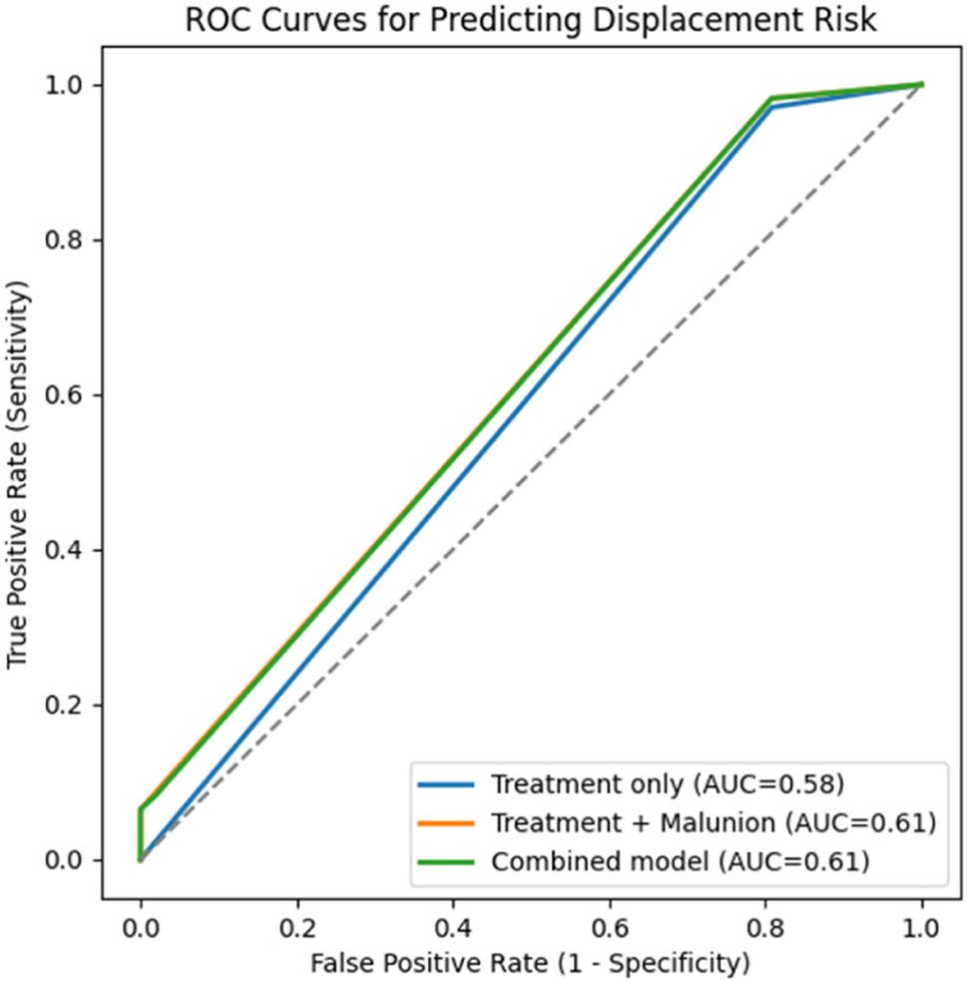
Receiver operating characteristic (ROC) curves for predicting fracture displacement

Funnel plot inspection suggested a low likelihood of publication bias (Fig. 8). However, interpretation should be cautious due to the small number of included studies.

## Discussion

This systematic review and meta‑analysis compared conservative and surgical management of minimally displaced (≤ 2 mm) humeral lateral condylar fractures (HLCF) in children. The pooled evidence indicated that conservative treatment was associated with a higher rate of secondary displacement compared with surgical fixation. Once fractures achieve union, long‑term outcomes—including elbow joint function, periarticular hyperplasia, and malunion—are broadly similar between conservative and surgical management. However, the decision to pursue conservative treatment inherently assumes that the fracture is truly stable, and whether this assumption is valid—and reliably identifiable based on minimal displacement alone—is precisely the question our study seeks to address.

Accurately assessing the severity and stability of minimally displaced HLCF remains challenging, as reflected by the wide variability in reported outcomes across studies [3, 15, 22–24]. This variability likely contributes to the substantial heterogeneity observed in the single‑arm analysis of conservative treatment. Differences in imaging protocols (e.g., AP, lateral, and oblique views), measurement techniques, immobilization strategies, follow‑up schedules, and thresholds for surgical conversion can all influence the apparent displacement and the decision to continue or abandon conservative management. These methodological and clinical differences provide a plausible explanation for the observed heterogeneity and also clarify why one study exerted disproportionate influence in the sensitivity analysis. Such variability is consistent with the divergent findings in the literature: while Marcheix et al. [3] and Bast et al. [15] reported excellent outcomes with conservative treatment, James et al. [22] and Franck et al. [13] documented substantially higher rates of secondary displacement and complications. Thomas et al. [24] further demonstrated that surgical fixation can achieve very low displacement rates, whereas delayed or inadequate management may increase the risk of malunion, growth disturbance, and other sequelae [25].

Variability in displacement measurement across studies may further contribute to inconsistent classification of “minimally displaced” fractures. The included studies used different radiographic views (AP, lateral, internal oblique, external oblique), measurement techniques, and classification systems, all of which introduce measurement error. Small differences in projection angle or ossification stage can alter the apparent fracture gap by more than 1–2 mm, potentially leading to misclassification of unstable fractures as “minimally displaced.” This variability likely contributes to the higher secondary displacement rates observed in conservatively treated patients.

Beyond case series, several studies have drawn preliminary conclusions. Salgueiro et al. [23] reported that, in fractures that later displace or in chronic cases, delayed treatment increases the difficulty of reduction, leads to greater surgical trauma and residual displacement, and is associated with higher rates of complications. Tejwani et al. [26] likewise noted that suboptimal management of HLCF often results in failure or deformity, underscoring the need for heightened clinical vigilance and ongoing radiographic evaluation.

The rationale for conservative treatment in pediatric fractures is grounded in children’s robust healing potential [27, 28]. For minimally displaced HLCF, successful non‑operative management likely depends on two key factors: (1) the integrity of the distal cartilaginous hinge and (2) the mechanical stability of the fracture following immobilization. These factors are often difficult to evaluate accurately; MRI can assist in assessing hinge integrity and thereby support treatment decision-making [29]. When both conditions are met, conservative treatment may yield favorable outcomes. Nonetheless, the lateral condyle is biomechanically vulnerable due to tensile forces from the extensor musculature and potential disruption of epiphyseal blood supply [30]. Additionally, prolonged exposure to synovial fluid may further impede fracture healing.

Given these findings, it is important to consider the certainty of the available evidence. According to the GRADE framework, the certainty of evidence for the primary outcome was rated as low. This reflects the predominance of retrospective study designs, potential selection bias, and imprecision related to low event rates rather than limitations of the present review. Evidence for secondary outcomes such as delayed healing and nerve injury was rated as very low, largely due to small sample sizes and inconsistent reporting across studies. Therefore, the findings of this review should be interpreted as associations rather than causal effects, and clinical recommendations should be made cautiously. High‑quality prospective studies are needed to strengthen the evidence base.

Although our findings show an association between conservative treatment and higher secondary displacement rates, the predominance of retrospective studies and the low certainty of evidence preclude causal inference. Therefore, recommendations for early surgical intervention should be made cautiously. Surgical fixation may be considered in cases with features suggestive of instability or in families wishing to minimize displacement risk, but individualized decision-making remains essential. Overall, management of minimally displaced (≤ 2 mm) HLCFs should integrate fracture characteristics, hinge integrity, imaging findings, the reliability of follow-up, and family preference rather than relying on displacement alone.

## Conclusions

Management of minimally displaced (≤ 2 mm) pediatric humeral lateral condylar fractures should be approached cautiously. Although conservative treatment is associated with a higher rate of secondary displacement, the certainty of evidence is low, and these findings should be interpreted as associations rather than causal effects. Close and structured radiographic follow-up remains essential for children managed non-operatively, with timely adjustment of treatment if displacement progresses. Treatment decisions should integrate displacement magnitude, fracture stability, imaging findings, follow-up reliability, and family preference to optimize outcomes.

## Limitations

This review has several limitations. First, there was an imbalance in the number of cases between the surgical and conservative groups, largely because existing literature on this fracture type disproportionately reports conservative management, which may affect the precision of between-group comparisons. Second, all included studies were observational, and the absence of randomized controlled trials limits the robustness and generalizability of the findings. Third, the certainty of evidence for most outcomes was rated as low or very low according to the GRADE framework, reflecting the inherent limitations of the available literature. Finally, although funnel plots were generated, the small number of included studies reduces the reliability of publication-bias assessment. High-quality prospective studies are needed to validate these findings and to better define selection criteria for conservative versus surgical care.

## Supplementary Information

Supplementary Material 1.

Supplementary Material 2.

Supplementary Material 3.

## Data Availability

All data generated or analysed during this study are included in this published article.
