# Supplementary material for: Surgical versus conservative management of minimally displaced (≤ 2 mm) pediatric lateral humeral condyle fractures: systematic review and meta-analysis
Source: BMC Musculoskelet Disord. 2026 Feb 14;27:232. doi: 10.1186/s12891-026-09555-w (PMC13011332; doi:10.1186/s12891-026-09555-w)
Supplement: Supplementary file 1 — Supplementary Material 1. [file 12891_2026_9555_MOESM1_ESM.docx]

**Electronic search strategies**

Database 1: PubMed (MEDLINE)

("Humeral Condyle"[Mesh] OR "lateral humeral condyle" OR "lateral condyle" OR "lateral humeral condyle fracture" OR "lateral condyle fracture")

AND

("Fractures, Bone"[Mesh] OR fracture* OR break*)

AND

(child* OR pediatric* OR paediatric* OR adolescen* OR infant* OR "young patient*" OR "skeletal immature")

AND

(displace* OR nondisplace* OR "minimally displaced" OR "≤2 mm" OR "2 mm")

AND

(conservative OR nonoperative OR non-operative OR cast OR immobilization OR surgery OR operative)

Database 2: Embase.

('lateral humeral condyle fracture'/exp OR 'lateral humeral condyle':ti,ab OR 'lateral condyle':ti,ab)

AND

('bone fracture'/exp OR fracture*:ti,ab)

AND

(child*:ti,ab OR pediatric*:ti,ab OR paediatric*:ti,ab OR adolescen*:ti,ab OR infant*:ti,ab)

AND

(displace*:ti,ab OR nondisplace*:ti,ab OR 'minimally displaced':ti,ab OR '≤2 mm':ti,ab)

AND

(conservative:ti,ab OR nonoperative:ti,ab OR 'non operative':ti,ab OR cast:ti,ab OR immobilization:ti,ab OR surgery:ti,ab)

Database 3: Cochrane Library

(lateral humeral condyle OR lateral condyle)

AND

(fracture OR bone injury)

AND

(child OR pediatric OR paediatric OR adolescent)

AND

(displaced OR nondisplaced OR minimally displaced)

AND

(conservative OR nonoperative OR cast OR surgery)

Database 4: Web of Science

TS = (

("lateral humeral condyle" OR "lateral condyle")

AND (fracture* OR break*)

AND (child* OR pediatric* OR paediatric* OR adolescen*)

AND (displace* OR nondisplace* OR "minimally displaced")

AND (conservative OR nonoperative OR cast OR surgery)

)
