# Supplementary material for: Surgical versus conservative management of minimally displaced (≤ 2 mm) pediatric lateral humeral condyle fractures: systematic review and meta-analysis
Source: BMC Musculoskelet Disord. 2026 Feb 14;27:232. doi: 10.1186/s12891-026-09555-w (PMC13011332; doi:10.1186/s12891-026-09555-w)
Supplement: Supplementary file 2 — Supplementary Material 2. [file 12891_2026_9555_MOESM2_ESM.docx]

Summary of Findings table

| **Outcome** | **No. of studies** | **Study design** | **Risk of bias** | **Inconsistency** | **Indirectness** | **Imprecision** | **Publication bias** | **Overall certainty** | **Summary of findings** |
| --- | --- | --- | --- | --- | --- | --- | --- | --- | --- |
| **Nonunion / secondary displacement** | 9 | Retrospective cohort | Serious (selection bias, incomplete follow‑up) | Serious (I² > 60%) | Not serious | Serious (low event rate) | Possible | **Low** | Conservative treatment associated with higher displacement risk, but causality cannot be established. |
| **Malunion** | 6 | Retrospective cohort | Serious | Not serious | Not serious | Serious | Possible | **Low** | Malunion occurred more frequently in conservative treatment, but absolute risk remained low. |
| **Delayed healing** | 5 | Retrospective cohort | Serious | Serious | Not serious | Serious | Possible | **Very low** | Evidence insufficient to determine whether conservative treatment increases delayed healing. |
| **Fracture healing rate** | 11 | Retrospective cohort | Serious | Not serious | Not serious | Serious | Possible | **Low** | Healing rate generally high (>90%) with conservative treatment. |
| **Nerve injury** | 3 | Retrospective cohort | Serious | Not serious | Not serious | Very serious (rare events) | Possible | **Very low** | No clear difference between treatments; evidence highly uncertain. |
